# Analysis of the Chemical Modification of Dental Enamel Submitted to 35% Hydrogen Peroxide “In-Office” Whitening, with or without Calcium

**DOI:** 10.1155/2017/4646789

**Published:** 2017-08-28

**Authors:** Rudá França Moreira, Fábio Pinheiro Santos, Estevão Antero Santos, Ramon Silva dos Santos, Marcelino José dos Anjos, Mauro Sayão de Miranda

**Affiliations:** ^1^School of Dentistry, UERJ, Rio de Janeiro, RJ, Brazil; ^2^Institute of Physics, UERJ, Rio de Janeiro, RJ, Brazil

## Abstract

**Purpose:**

The purpose of this study was to evaluate changes in calcium and phosphorus content in dental enamel when subjected to “in-office” whitening for an extended time by using a 35% hydrogen peroxide solution, with and without calcium.

**Materials and Methods:**

10 human teeth, from which the roots had been removed, were embedded in epoxy resin, and their surfaces were smoothed. The specimens were divided into two groups; in group 1, a whitening solution without calcium was used, while in group 2, the solution included calcium. Each specimen was evaluated at 6 different points before the bleaching treatment, and these points were reassessed after each session. A total of five sessions were carried out. Concentrations of calcium and phosphorus were measured by using the technique of X-ray fluorescence.

**Results:**

After performing a statistical analysis, it was found that there was no statistically significant loss of calcium and phosphorus during the whitening treatment, and the groups showed no statistical differences.

**Conclusion:**

Excessive use of hydrogen peroxide, with or without calcium, causes no loss of calcium and phosphorus.

## 1. Introduction

Tooth whitening has become one of the most used techniques in dental clinics [1], and this is due to the characteristics of this technique, which presents quickly noninvasive aesthetic results and does not require anesthesia and the use of drill motors and bits [2, 3]. For vital teeth, tooth whitening can be performed with a supervised at-home technique by using a solution of 3% to 10% hydrogen peroxide and 10% to 22% carbamide peroxide; by using the “in-office” technique using a solution of 35% to 38% hydrogen peroxide and 35% carbamide peroxide; or by using the mixed technique that is a mixture of the aforementioned techniques. The treatment time depends on concentration and formulation from the bleaching agent, as well as the regime; it is believed that highly concentrated bleaching agents give satisfactory results in shorter periods that may vary from 4 to 6 weeks, regardless of the whitening agent used in home and office therapy [4].

Despite all the related advantages and antimicrobial potential of whitening agents [5], care must be taken because, according to some authors, there are some side effects, such as increased roughness, mineral loss, reduction of mechanical properties of dental tissue, a cocarcinogenic effect [6–8], and decrease in the strength values of direct restorative materials [9, 10]. Variations in the concentration of whitening agents may alter the intensity of the loss of mineral content of dental tissues, but this side effect is still controversial in the literature [11].

In attempt to minimize the risks of demineralization of dental structures, manufacturers have sought changes in the chemical formulations of their bleaching agents. One of these changes was the introduction of calcium ions. Teeth that undergo a dental whitening technique, with absence of calcium, have a greater change of microhardness in the enamel than the whitening agents that have associated calcium. Calcium association with a higher hydrogen peroxide concentration may decrease microhardness changes on enamel [12].

Whitening over an extended period of time is usually employed by dental surgeons who are seeking better results in their clinical practice [13] or in cases of severe staining by medications, especially tetracycline, which often require a long time in whitening therapy [14].

Evaluating inorganic matter in enamel by the technique of X-ray fluorescence (XRF) has advantages over other techniques, as it can accurately be used to analyze the amount of calcium and phosphorus, as well as being a nondestructive technique. Thus, it is possible to perform various readings of the same specimen, always at the exact spot where the first evaluation was performed, and in different areas of the same specimen and at various times in whitening therapy [15–17].

The aim of this study was to evaluate, by using the technique of XRF, changes in calcium and phosphorus content in the enamel submitted to whitening treatment for extended periods and whitening treatments with hydrogen peroxide gels, with and without calcium in the formulation thereof.

## 2. Materials and Methods

In compliance with Resolution 466/2012 of the National Health Council (*Conselho Nacional de Saúde* (CNS)), the study was submitted for consideration by the Committee of Ethics in Research (*Comitê de Ética em Pesquisa* (CEP)) of the Pedro Ernesto University Hospital (*Hospital Universitário Pedro Ernesto* (HUPE)), and University of the State of Rio de Janeiro (*University do Estado do Rio de Janeiro* (UERJ)), and it was approved under number 16076913.3.0000.5259.

Ten upper anterior human teeth were used. The samples were stored in a 0.05% thymol solution for up to one week before conducting the tests. The cleaning was done by scraping the outer surface with periodontal curettes and prophylaxis with pumice and water, with the aid of a rubber cup, a micromotor, and a contra-angle handpiece.

The teeth were sectioned at the cementoenamel junction, discarding the root portion, and they were stored in ultrapure water at 4°C. Then the teeth were affixed in epoxy resin with the aid of a dental parallelometer so that the labial surface was parallel to the horizontal plane of the bench and they were secured in a special Teflon device, which was attached to an articulated platform with movements along the *x*, *y*, and *z* directions so that the area analyzed by XRF was always in the same position.

The tooth set/base was taken to a metallographic polisher (AROTEC®, model APL-4), employing 600-grit silicon carbide sandpaper and rinsing it well, and thus standardization was done by smoothing the enamel surface. After inclusion and smoothing, the specimens (CP) were immersed for 5 minutes in an ultrasonic bath (ultrasonic tank, UltraCleaner 700, Unique®, Indaiatuba, SP, Brazil).

The specimens were divided into two groups (5 specimens per group):

  Group 1: 35% Whiteness HP Maxx (FGM, Brazil) and 35% hydrogen peroxide
  Group 2: 35% Whiteness HP Blue (FGM, Brazil) and 35% hydrogen peroxide with calcium

They were manipulated and applied by following the manufacturers' guidelines. In group 1, at each session, three fifteen-minute applications of gel were performed, while in group 2, one application was conducted for 45 minutes. Five sessions were held for each group, and the specimens were stored in ultra-pure water between sessions.

The evaluations were performed before bleaching therapy (baseline) and after each session by using the technique of XRF. The specimens were evaluated six times at different points. During the first scanning, these points had their coordinates (*x*, *y*) fixed so that subsequent readings were performed in exactly the same positions.

**Figure 1:**
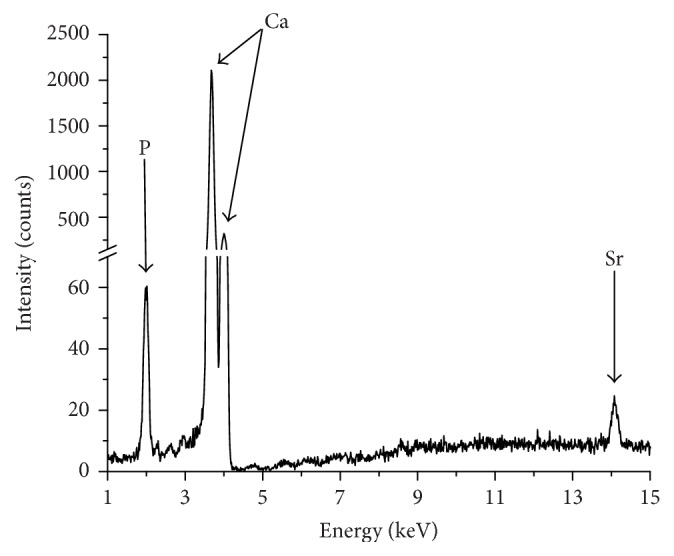
X-ray fluorescence spectrum of tooth enamel.

Measurements by XRF generated spectrographic data, which represent the signatures of the chemical elements present in the sample.  Figure 1 shows a spectrum of XRF and detected the following elements: P, Ca, and Sr.

## 3. Results

**Figure 2:**
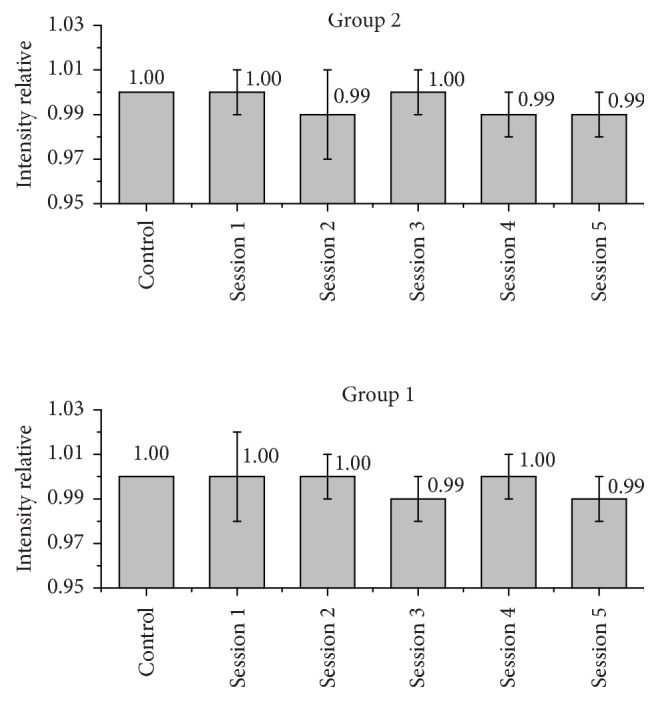
Relative intensity of calcium in the groups that were evaluated.

**Figure 3:**
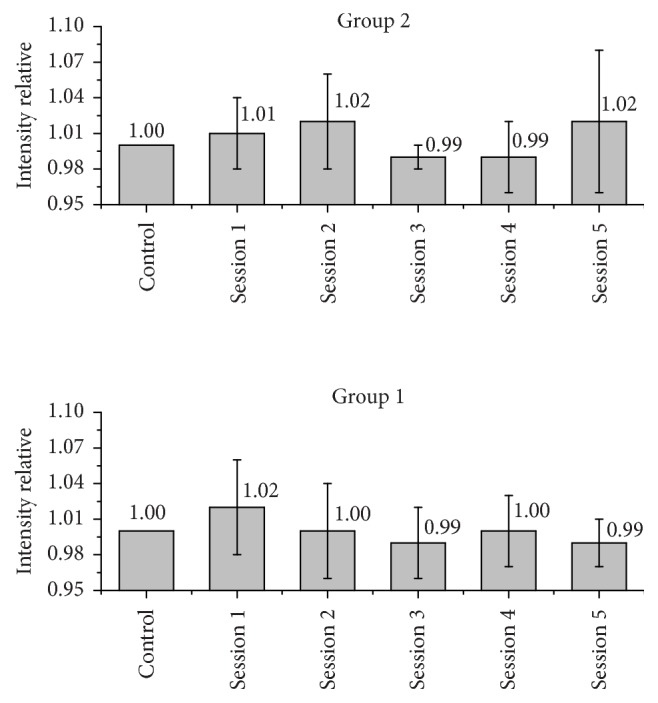
Relative intensity of phosphorus in the groups that were evaluated.

Figures 2 and 3 represent the variations in the counts of Ca and P per group, after application of the respective bleaching gels at each treatment. The relative intensity was obtained by normalizing each XRF measurement in a direct relationship with the baseline.

(1) $$\begin{array}{c} I_{\text{relative}}=\frac{I_{\text{session }x}}{I_{\text{baseline}}}. \end{array}$$

In this equation, *I*_baseline_ is equivalent to the initial count of the specimens and *I*_session  *x*_ is equivalent to measurements obtained after whitening sessions, and “*x*” is the number of whitening sessions to which the tooth was exposed.

Figure 2 shows the changes that occurred in the calcium content of the enamel, and Figure 3 shows the changes in phosphorus count of the groups tested.

From (1), it can be noted that the results less than 1.0 indicate a reduction of mineral concentrations, while results above 1.0 indicate an increase of mineral content.

The Bonferroni test was used initially for parametric comparison of average losses of Ca and P at each stage of treatment within groups with a significance level of 5%.

No statistically significant differences (*a* < 0.05) were found between the average counts of calcium and phosphorus of all the readings that were taken (baseline, session 1, session 2, session 3, session 4, and session 5).

Student's *t*-test was used to compare measurements of groups of different treatments, with a minimum significance level of 5%. It was observed that there is no statistical difference between the groups after five sessions (*a* > 0.05).

## 4. Discussion

Several side effects caused by whitening vital teeth have been reported in the literature, and the one that is most often cited is dental sensitivity [6, 11, 18–20], but there are others, such as a change in mechanical properties of enamel, dentin, and direct restorative materials [6, 8, 9, 21–23], an increase in roughness of these tissues [1, 8, 13, 24–30], and change in mineral content [1, 12, 20, 25, 29–32].

These side effects have not yet been fully explained in dental literature [6], especially regarding the impact of hydrogen peroxide on mineral content of dental tissues.

A literature review in 2007 [6] showed that, as of the date of publication, the studies were almost unanimous in stating that there was no change in mineral content in the enamel surface. However, many studies performed after that date have shown the opposite [1, 13, 21, 26, 30–33]. This is due to the wide variety of methodologies, without there being a well-defined protocol for this type of study, and the use of nonideal tests to measure loss of calcium and phosphorous in tooth enamel.

Scanning electronic microscopy (SEM) and energy dispersive X-ray detector (EDS) are widely used methods because they are quick and convenient tests for the morphological and semiquantitative analysis of the surface of enamel samples that have been subjected to whitening [6]. Nonetheless, because they are destructive methods, they do not allow a comparison by using the same specimen, which can cause incorrect and inconclusive analysis. Evaluation by SEM using a low amount of vacuum is not destructive, but it presents a lower degree of acuity and is little used in studies of changes in the mineral content of teeth [1, 13, 30, 33].

Morphological evaluation by Atomic Force Microscopy (AFM), according to Pedreira de Freitas et al. (2010) [24] and Zimmerman et al. (2010) [25], is the most suitable technique to analyze changes in human enamel, but quantitative evaluations are necessary to complement this type of study.

The methodology of microhardness is also widely used to measure mineral content in the tooth structure, because there is a direct relationship between the hardness and mineral content of the specimen [6, 12, 23], but other studies have shown that the loss of organic content caused by use of peroxides significantly alters the mechanical properties and the microhardness of the material tested [24, 25, 34] has shown that microhardness and mineral content of enamel and dentin were decreased by bleaching treatment in human third molars, although their study revealed that these properties were positively affected by two-week postoperative treatment with artificial saliva and amorphous calcium phosphate.

Another methodology that is used, albeit on a smaller scale, is the study of the density of the material, which is a ratio of mass per volume that is measured in two stages, before and after whitening therapy [31]. In this study, there was no way to evaluate which tissue was lost (organic or inorganic); therefore, changes detected in this test do not truly constitute demineralization.

The results of this study demonstrate that there is no demineralization of the enamel surface when subjected to clinical whitening with different whitening agents, which corroborates with the authors studied by Joiner in 2007 [6], but they differ from Cakir et al. (2011) [1]; Souza et al. (2010) [13]; Al-Salehi et al. (2007) [21]; Gjorgievska and Nicholson (2011) [26]; González-López et al. (2016) [30]; Efeoglu et al. (2007) [31]; Jiang et al. (2008) [32]; Poorni et al. (2010) [33]; and Klaric et al. (2015) [34].

This difference is due to the methodology used, as it is possible to evaluate the same specimen several times and at various stages, which allows a more accurate comparison than those obtained in other tests.

As demineralization was not detected in this study, the increase in roughness that the enamel underwent, which was stated by Cakir et al. (2011) [1]; Izquierdo-Barba et al. (2015) [8]; Souza et al. (2010) [13]; Pedreira de Freitas et al. (2010) [24]; Zimmerman et al. (2010) [25]; Gjorgievska and Nicholson (2011) [26]; Navimipour et al. (2012) [27]; Navimipour et al. (2013) [28]; and Sa et al. (2013) [29], is probably due to the property of hydrogen peroxide that denatures proteins which are present in the region of interprismatic enamel, creating valleys, as described by Pedreira de Freitas et al. (2010) [24] and Zimmerman et al. (2010) [25].

The presence of calcium in the formulation of the gels did not alter the mineral content of tooth enamel, and this can be explained by the fact that there was no demineralization of the tissue, and therefore there was no remineralization.

The specimens were not stored in saliva, as Moreira et al. (2014) [35] had showed that there was no statistical difference between whitened teeth that were stored in distilled water and those stored in artificial saliva, because of the remineralization potential of saliva, which can change the results, which presents one more variable [36].

## 5. Conclusion

Within the limitations of this study, it was possible to conclude the following:

1. The whitening gels that were tested showed no potential of modifying the content of calcium and phosphate of human dental enamel.
2. Even when whitening gels were used excessively, there was no demineralization of tooth enamel.
